# Impacts of Digital Healthy Diet Literacy and Healthy Eating Behavior on Fear of COVID-19, Changes in Mental Health, and Health-Related Quality of Life among Front-Line Health Care Workers

**DOI:** 10.3390/nu13082656

**Published:** 2021-07-30

**Authors:** Dinh N. Vu, Dung T. Phan, Hoang C. Nguyen, Lan T. H. Le, Huu C. Nguyen, Tung H. Ha, Hung K. Dao, Manh V. Trinh, Thinh V. Do, Hung Q. Nguyen, Thao T. P. Nguyen, Thuy T. Le, Cuong Q. Tran, Khanh V. Tran, Trang T. Duong, Hai X. Pham, Vinh-Tuyen T. Le, Tuyen Van Duong

**Affiliations:** 1 Director Office, Military Hospital 103, Hanoi 121-08, Vietnam; vunhatdinh@vmmu.edu.vn; 2 Department of Trauma and Orthopedic Surgery, Vietnam Military Medical University, Hanoi 121-08, Vietnam; 3 Nursing Office, Thien An Obstetrics and Gynecology Hospital, Hanoi 112-06, Vietnam; phanthidzungvd@gmail.com; 4 Faculty of Nursing, Hanoi University of Business and Technology, Hanoi 116-22, Vietnam; 5 Director Office, Thai Nguyen National Hospital, Thai Nguyen City 241-24, Vietnam; nguyenconghoang@tnmc.edu.vn (H.C.N.); lanhuong.bvtutn@gmail.com (L.T.H.L.); 6 President Office, Thai Nguyen University of Medicine and Pharmacy, Thai Nguyen City 241-17, Vietnam; 7 Training and Direction of Healthcare Activity Center, Thai Nguyen National Hospital, Thai Nguyen City 241-24, Vietnam; 8 Biochemistry Department, Thai Nguyen National Hospital, Thai Nguyen City 241-24, Vietnam; 9 Director Office, E Hospital, Hanoi 113-08, Vietnam; bacsyhuu@gmail.com; 10 Department of Thoracic and Cardiovascular Surgery, E Hospital, Hanoi 113-08, Vietnam; 11 Director Office, General Hospital of Agricultural, Hanoi 125-16, Vietnam; hahuutung.200564@gmail.com; 12 Director Office, Bac Ninh Obstetrics and Pediatrics Hospital, Bac Ninh 161-23, Vietnam; daokhachung2000@yahoo.com; 13 Director Office, Quang Ninh General Hospital, Quang Ninh 011-08, Vietnam; trinhmanhqnsyt@gmail.com; 14 Director Office, Bai Chay Hospital, Quang Ninh 011-21, Vietnam; dovanthinhhscc@gmail.com; 15 Director Office, Quang Ninh Obstetrics and Pediatrics Hospital, Quang Ninh 011-24, Vietnam; bshungbvqn@gmail.com; 16 Health Management Training Institute, University of Medicine and Pharmacy, Hue University, Thua Thien Hue 491-20, Vietnam; nguyenthiphuongthao@hueuni.edu.vn; 17 Department of Health Economics, Corvinus University of Budapest, 1093 Budapest, Hungary; 18 Faculty of Medical Laboratory Science, Da Nang University of Medical Technology and Pharmacy, Da Nang 502-06, Vietnam; ltthuy@dhktyduocdn.edu.vn; 19 President Office, Da Nang University of Medical Technology and Pharmacy, Da Nang 502-06, Vietnam; 20 Director Office, Thu Duc City Health Center, Ho Chi Minh City 713-10, Vietnam; quoccuong.mph@gmail.com (C.Q.T.); phamxuanhai1976@gmail.com (H.X.P.); 21 Faculty of Health, Mekong University, Vinh Long 852-16, Vietnam; 22 Director Office, Le Van Thinh Hospital (Previously Hospital District 2), Ho Chi Minh City 711-13, Vietnam; drtvkhanh.bvq2@gmail.com; 23 Nursing Office, Tan Phu District Hospital, Ho Chi Minh City 720-16, Vietnam; duongtrang7273@gmail.com; 24 Department of Pharmacognosy-Traditional Pharmacy-Pharmaceutical Botanic, Can Tho University of Medicine and Pharmacy, Can Tho 941-17, Vietnam; ltvtuyen@ctump.edu.vn; 25 Ph.D. Program in Clinical Drug Development of Herbal Medicine, College of Pharmacy, Taipei Medical University, Taipei 110-31, Taiwan; 26 School of Nutrition and Health Sciences, Taipei Medical University, Taipei 110-31, Taiwan

**Keywords:** digital healthy diet literacy, healthy eating behavior, fear of COVID-19, mental health, health-related quality of life, front-line health care workers, symptoms like COVID-19, epidemic containment experience, health literacy, Vietnam

## Abstract

Background: We aimed to examine the impacts of digital healthy diet literacy (DDL) and healthy eating behaviors (HES) on fear of COVID-19, changes in mental health, and health-related quality of life (HRQoL) among front-line healthcare workers (HCWs). Methods: An online survey was conducted at 15 hospitals and health centers from 6–19 April 2020. Data of 2299 front-line HCWs were analyzed—including socio-demographics, symptoms like COVID-19, health literacy, eHealth literacy, DDL, HES, fear of COVID-19, changes in mental health, and HRQoL. Regression models were used to examine the associations. Results: HCWs with higher scores of DDL and HES had lower scores of FCoV-19S (regression coefficient, B, −0.04; 95% confidence interval, 95% CI, −0.07, −0.02; *p* = 0.001; and B, −0.10; 95% CI, −0.15, −0.06; *p* < 0.001); had a higher likelihood of stable or better mental health status (odds ratio, OR, 1.02; 95% CI, 1.00, 1.05; *p* = 0.029; and OR, 1.04; 95% CI, 1.00, 1.07; *p* = 0.043); and HRQoL (OR, 1.02; 95% CI, 1.01, 1.03; *p* = 0.006; and OR, 1.04; 95% CI, 1.02, 1.06; *p* = 0.001), respectively. Conclusions: DDL and HES were found as independent predictors of fear of COVID−19, changes in mental health status, and HRQoL in front-line HCWs. Improving DDL and HES should be considered as a strategic approach for hospitals and healthcare systems.

## 1. Introduction

The burden of mental health problems among healthcare workers (HCWs) has been highlighted, especially in low and middle-income countries [1]. The high prevalence of psychological health problems in HCWs was reported in meta-analyses [2,3,4,5]. The problem has similarly been reported in Vietnam during the COVID-19 pandemic [6].

HCWs face social stigma, discrimination, raising concerns about their safety and family members’ health [7,8,9]. Front-line HCWs are even at high risk of COVID-19 infection [10]. These affect their psychological health and well-being [7,8,9], especially when they do not feel protected [11]. Therefore, their fear reaction, changes in mental health, and health-related quality of life during the pandemic should be addressed. It suggests developing early evidence-based sustainable interventions to prevent and mitigate the short- and long-term effect of the COVID-19 pandemic on HCWs’ mental health and well-being [12,13,14,15]. The improvement of psychological health and well-being could enhance the infection control practices among HCWs [16], which may further improve patient care quality [17]. Therefore, we must address the impact of facing COVID-19 in HCWs on their psychological health and well-being and identify the associated predictors which might further contain the pandemic.

The symptoms like COVID-19 negatively influenced on people’s mental health and health-related quality of life (HRQOL) [18]. Digital healthy diet literacy (DDL) was linked with healthier eating behavior [19] which further modified the negative effect of COVID-19 on mental health [20]. In addition, health literacy, eHealth literacy (eHEALS), and DDL significantly modified the negative impact of COVID-19 on HRQOL [21].

The roles of DDL and healthy eating behaviors (HES) on mental health and quality of life have been investigated in patients and students [19,20]. However, the roles of DDL and HES have not been studied on HCWs. In addition, their role in fear was also remained to be explored. Therefore, we aimed to examine the associated predictors of fear of COVID-19, changes in mental health, and health-related quality of life (HRQoL) among front-line HCWs, in which impacts of DDL and HES were emphasized.

## 2. Materials and Methods

### 2.1. Study Design and Participants

A cross-sectional study was approved by the Ethical Review Committee of Hanoi University of Public Health, Vietnam (IRB no. 133/2020/YTCC-HD3). The informed consent was achieved from all participants before taking the survey.

An online survey was conducted from 6–19 April 2020 on HCWs who were working at 12 hospitals and three health centers across Vietnam [22]. We analyzed a sample of 2299 front-line HCWs (who were working at the outpatient department, emergency department, isolation areas, imaging and laboratory diagnosis department, patient administration areas), including 90 from Military Hospital 103; 142 from E hospital; 186 from General Hospital of Agricultural; 369 from Thai Nguyen National Hospital; 142 from Bac Ninh Obstetrics and Pediatrics Hospital; 249 from Quang Ninh General Hospital; 187 from Bai Chay Hospital; 143 from Quang Ninh Obstetrics and Pediatrics Hospital; 137 from Trieu Phong District Health Center; 11 from Da Nang Oncology Hospital; 134 from Tan Phu District Hospital; 169 from Le Van Thinh Hospital (previously Hospital District 2); 78 from District 9 Health Center (now joined with the Thu Duc City Health Center); 155 from Thu Duc City Health Center (previously the Thu Duc District Health Center); 107 from Can Tho University of Medicine and Pharmacy Hospital.

### 2.2. Measurements

#### 2.2.1. Participants’ Characteristics

The first part of the questionnaire was to ask HCWs about their age, gender, marital status, ability to pay for medication, self-perceived social status, types of health care personnel (doctors, nurses, or others), and previous epidemic containment experiences (e.g., SARS, tuberculosis, influenza A). Body mass index (BMI, kg/m^2^) was calculated by weight (kg)/height (m^2^). In addition, comorbid conditions were assessed using the items of the Charlson comorbidity index [23,24].

#### 2.2.2. Symptoms like COVID-19

The next part was to ask HCWs about the symptoms that were similar to COVID-19 symptoms (S-COVID-19-S) [25], including fever, cough, and dyspnea, myalgia, fatigue, sputum production, confusion, headache, sore throat, rhinorrhea, chest pain, hemoptysis, diarrhea, and nausea/vomiting. HCWs were classified as having S-COVID-19-S if they had any of those symptoms. The S-COVID-19-S showed the negative impact on mental health and HRQOL in previous studies [18,26].

#### 2.2.3. Health Literacy and Digital Healthy Diet Literacy

Health literacy (HL) and digital healthy diet literacy (DDL) were assessed using a 12-item short-form health literacy questionnaire (HLS-SF12) [27,28], and four-item digital healthy diet literacy (DDL-4) [19]. These tools were validated and used in the Vietnamese context [19,21]. HCWs responded to each item on a four-point Likert scale from 1 (very difficult) to 4 (very easy). HL index and DDL index scores were standardized to a unified metric from 0 to 50, using formula (1), with a higher score indicating better HL [19,29]

*Index* = (*Mean* − *1*) × (*50*/*3*) (1)

where *Index* is the specific index calculated, *Mean* is the mean of all participating items (12 items for HL index, 4 items for DDL index), *1* is the minimal possible mean (leading to a minimum index of 0), *3* is the range of the mean, and *50* is the chosen maximum index value.

#### 2.2.4. eHealth Literacy Scale

HCWs’ eHealth literacy was assessed using the eHealth literacy scale (eHEALS) with eight items [30] which was validated and used in Vietnam [21,22]. HCWs reported their experiences on a five-point Likert scale from 1 (strongly disagree) to 5 (strongly agree). The sum scores range from 8 to 40, with high scores indicating better eHEALS.

#### 2.2.5. Healthy Eating Behavior

Healthy eating behavior was assessed using the 5-item healthy eating score (HES-5) [31,32]. The HES-5 utilization was comparable with the 2015 health eating index in assessing the overall diet quality [32] which was validated and used in Vietnam [20]. HCWs reported their eating/drinking frequency of fruits, vegetables, whole grains, dairy, and fish over the last 30 days on a five-point scale from 0 (rarely or never) to 1 (1–2 times/week), 2 (3–6 times/week), 3 (once/day), 4 (twice/day), and 5 (3 or more times/day). The total HES scores range from 0 to 25, the higher score the better healthy eating behavior.

#### 2.2.6. Fear of COVID-19

The fear was assessed using the seven-item fear of COVID-19 scale (FCoV-19S) [33] which was used in Vietnam [21,34]. HCWs responded to each item on a five-point Likert scale from 1 (strongly disagree) to 5 (strongly agree). The sum score of seven items ranges from 7 to 35, with higher scores indicating greater fear.

#### 2.2.7. Changes in Mental Health and Health-Related Quality of Life

Healthcare workers (HCWs), in fact, have better knowledge and ability to detect the changes in their mental health and HRQOL than the general public. Therefore, to assess the changes, HCWs were asked using the question “All in all, how could you say your mental health currently is, as compared with that before the pandemic?”, and “All in all, how could you say your health-related quality of life (HRQoL) is, as compared with that before the pandemic?”. The response options were (1) “worse”, (2) “stable”, or (3) “better”. To facilitate the analysis, we classified the responses into two groups (worse vs. stable or better).

### 2.3. Data Collection Procedure

Investigators and research assistants (doctors, nurses, medical students) of each hospital sent to survey link to front-line HCWs via messenger or email. The contact information was available from human resource office of each hospital. HCWs had signed the online informed consent before took the survey. It took about 15–20 min to complete the survey. The mandatory fields were applied for all survey questions to eliminate the missing data. Data were coded and analyzed confidentially by researchers.

### 2.4. Data Analysis

Firstly, one-way ANOVA test and chi-square test were used appropriately to explore the distributions of FCoV-19S, changes in mental health, and HRQoL by different categories of independent variables. Secondly, bivariate and multivariate linear regression models and logistic regression models were used to examine the associated factors of FCoV-19S, changes in mental health, and HRQoL. To minimize residual effects of confounders, all the factors in the bivariate model were analyzed in the multivariate model [35]. To eliminate the multicollinearity in multivariate models, the correlations among independent variables were analyzed using Spearman correlation. Since the DDL moderately correlates with HL (*rho* = 0.68) and eHEALS (*rho* = 0.38; Table S1), DDL and other independent variables were analyzed in the multivariate models. Data were analyzed using the IBM SPSS Version 20.0 for Windows (IBM Corp, Armonk, NY, USA). The significance level was set at a *p*-value < 0.05.

## 3. Results

### 3.1. Characteristics of Front-Line HCWs

In 2299 HCWs, 19.9% were aged 41–60, 33.2% were men, 26.0% were doctors, 41.1% were nurses, 32.9% were other types of health care personnel, 43.8% had experiences in containing previous epidemics, 14.7% were with S-COVID-19-S. The means of FCoV-19S, HL, eHEALS, DDL, and HES were 18.8 ± 5.3, 36.1 ± 7.1, 33.1 ± 4.8, 35.8 ± 8.2, and 14.6 ± 4.8, respectively. The proportion of HCWs with stable or better mental health status was 93.5% (2150/2299), and those with stable or better HRQoL were 80.8% (1858/2299) (Table 1).

### 3.2. Associated Factors of Fear of COVID-19

In the multivariate analysis, men (regression coefficient, B, −0.72; 95% confidence interval, 95% CI, −1.20, −0.24; *p* = 0.003), doctors (B, −1.63; 95% CI, −2.20, −1.06; *p* < 0.001), and those with higher scores of DDL (B, −0.04; 95% CI, −0.07, −0.02; *p* = 0.001), and HES (B, −0.10; 95% CI, −0.15, −0.06; *p* < 0.001) had lower scores of FCoV-19S. Whereas, HCWs who had been ever married had a higher score of FCoV-19S (B, 0.88; 95% CI, 0.36, 1.39; *p* = 0.001), as compared to their counterparts (Table 2).

### 3.3. Associated Factors of Mental Health Changes

In the multivariate analysis, HCWs had a higher likelihood of stable or better mental health were those with experiences in containing previous epidemics (odds ratio, OR 1.62; 95% CI, 1.11, 2.35; *p* = 0.012), with higher scores of DDL (OR, 1.02; 95% CI, 1.00, 1.05; *p* = 0.029), and HES (OR, 1.04; 95% CI, 1.00, 1.07; *p* = 0.043). Whereas, HCWs with S-COVID-19-S had a lower likelihood of stable or better mental health (OR, 0.36; 95% CI, 0.25, 0.53; *p* < 0.001), as compared to their counterparts (Table 3).

### 3.4. Associated Factors of HRQoL Changes

In the multivariate analysis, HCWs had a higher likelihood of stable or better HRQoL were those aged 41–60 years (OR, 1.53; 95% CI, 1.13, 2.07; *p* = 0.006), those very or fairly easily paid for their medication (OR, 1.43; 95% CI, 1.14, 1.78; *p* = 0.002), those with middle or high social status (OR, 1.37; 95% CI, 1.02, 1.84; *p* = 0.037), those with experiences in containing previous epidemics (OR 1.29; 95% CI, 1.03, 1.61; *p* = 0.026), with higher scores of DDL (OR, 1.02; 95% CI, 1.01, 1.03; *p* = 0.006), and HES (OR, 1.04; 95% CI, 1.02, 1.06; *p* = 0.001). Whereas, HCWs had a lower likelihood of stable or better HRQoL were men (OR, 0.76; 95% CI, 0.60, 0.97; *p* = 0.024), and those with S-COVID-19-S had a lower likelihood of stable or better mental health (OR, 0.53; 95% CI, 0.40, 0.69; *p* < 0.001), as compared to their counterparts (Table 4).

## 4. Discussion

Results of the current study highlight the associations of digital healthy diet literacy (DDL) and healthy eating behavior (HES) with fear of COVID-19 (FCoV-19S), changes in mental health, and health-related quality of life (HRQoL).

In the current study, higher scores of DDL and HES were associated with a higher likelihood of having stable or better mental health status and HRQoL during the pandemic. The link between eHEALS and DDL was also found in this study. They both showed the benefits on HRQOL during the pandemic as reported in a previous study [21]. In addition, DDL was linked with higher likelihood of healthier eating behavior in nursing and medical students [19]. In turn, healthy eating behavior showed a beneficial impact on depression in outpatients [20], on depression, and quality of life in college students [36]. Fruits and vegetables showed positive impacts on mental health in the adult population [37]. Moreover, healthy eating behavior was also associated with a higher score of well-being in HCWs [38]. Therefore, improving HCWs’ DDL and providing healthy meals are the important responsibility of managers, policymakers which could significantly improve HCWs’ mental health and HRQoL life amidst the pandemic. Improving DDL and HES should be added in the global actions to protect HCWs from negative effects of the COVID-19 pandemic [39].

Both DDL and HES were associated with lower FCoV-19S scores in HCWs in the current study. This finding is in light of previous evidence that people with higher health literacy had lower fear scores [34]. Healthy dietary habits were associated with lower depression states in college students [36]. In addition, healthy food habits were also linked to better psychological health during the pandemic [40]. HCWs are not immune to fear and may suffer from a higher rate of fear than others, especially front-line HCWs. Therefore, courage is a decision to enhance DDL, and HES, and in turn, reduce fear, and adhere to infection prevention practices to provide a higher quality of care [41].

Besides, as compared to HCWs with no previous epidemic containment experience, those with the experiences had a higher likelihood of stable or better mental health status and HRQoL. In addition, doctors had lower FCoV-19S scores. This could be explained that experienced HCWs have a better ability to regulate their negative emotions, and maintain their quality of life. In addition, male HCWs had lower FCoV-19S scores but worse HRQoL during the pandemic. A previous study has reported that female HCWs are more vulnerable to mental health problems than males [42]. Male HCWs reported a lower fear of COVID-19 than females [43].

In addition, front-line HCWs with ever married status had a higher FCoV-19S score. This could be linked with HCWs’ concerns about their own and family members’ safety and health which made them feel a greater fear of COVID-19 [7,8].

Lastly, HCWs aged 41–60 years, or those with better ability to pay for medication had a higher likelihood of stable or better HRQoL during the pandemic. This could be explained that HCWs at the age of 41–60 years usually had a better financial status which may help to facilitate their life better and keep quality of life stable during the pandemic. The older age was also found to be associated with better well-being in HCWs in a previous study [38].

Our study has some limitations. First, the reporting bias might happen in measuring the changes in mental health and HRQoL states. However, the studied population is HCWs which could minimize the bias. Second, COVID-19 infection may influence the results as we cannot identify the positive COVID-19 cases. The results can be guaranteed as no new confirmed case in HCWs during the survey time [44]. Third, the causal relationship cannot be drawn from a cross-sectional design. Despite the mentioned drawbacks, the findings could help to suggest potential research directions and interventions that improve HCWs’ mental health and quality of life.

## 5. Conclusions

Digital healthy diet literacy (DDL) and healthy eating behaviors (HES) were found as independent predictors of fear of COVID-19, changes in mental health status, and health-related quality of life (HRQoL) in front-line healthcare workers (HCWs). A strategic approach to improve DDL and HES is highly suggested to protect HCWs’ psychological health and quality of life during the pandemic, especially for front-line HCWs.

## Figures and Tables

**Table 1:** Characteristics, fear of COVID-19, changes in mental health, and health-related quality of life among front-line health care workers.

Variables	Total (*n* = 2299)	FCoV-19S		Worse MH * (*n* = 149)	Stable or Better MH * (*n* = 2150)		Worse HRQoL ** (*n* = 441)	Stable or Better HRQoL ** (*n* = 1858)	
	*n* (%)	Mean ± SD	*p*	*n* (%)	*n* (%)	*p*	*n* (%)	*n* (%)	*p*
Age, year			0.444			0.160			0.004
21–40	1842 (80.1)	18.7 ± 5.2		126 (84.6)	1716 (79.8)		375 (85.0)	1467 (79.0)	
41–60	457 (19.9)	18.9 ± 5.3		23 (15.4)	434 (20.2)		66 (15.0)	391 (21.0)	
Gender			<0.001			0.324			0.003
Women	1535 (66.8)	19.1 ± 5.1		94 (63.1)	1441 (67.0)		268 (60.8)	1267 (68.2)	
Men	764 (33.2)	18 ± 5.6		55 (36.9)	709 (33.0)		173 (39.2)	591 (31.8)	
Marital status			0.001			0.077			0.705
Never married	542 (23.6)	18.1 ± 5.3		44 (29.5)	498 (23.2)		107 (24.3)	435 (23.4)	
Ever married	1757 (76.4)	19 ± 5.2		105 (70.5)	1652 (76.8)		334 (75.7)	1423 (76.6)	
Ability to pay for medication			0.004			0.033			<0.001
Very or fairly difficult	1210 (52.6)	19.1 ± 5.5		91 (61.1)	1119 (52.0)		269 (61.0)	941 (50.6)	
Very or fairly easy	1089 (47.4)	18.4 ± 5		58 (38.9)	1031 (48.0)		172 (39.0)	917 (49.4)	
Social status			0.084			0.044			0.001
Low	307 (13.4)	19.3 ± 5.9		28 (18.8)	279 (13.0)		80 (18.1)	227 (12.2)	
Middle or high	1992 (86.6)	18.7 ± 5.1		121 (81.2)	1871 (87.0)		361 (81.9)	1631 (87.8)	
Type of health care personnel			<0.001			0.267			0.804
Others	756 (32.9)	19.3 ± 5.4		47 (31.5)	709 (33.0)		144 (32.7)	612 (32.9)	
Nurse	945 (41.1)	19.2 ± 5.1		55 (36.9)	890 (41.4)		177 (40.1)	768 (41.3)	
Doctor	598 (26.0)	17.4 ± 5.1		47 (31.5)	551 (25.6)		120 (27.2)	478 (25.7)	
Epidemic containment experience			0.188			0.001			0.004
No	1292 (56.2)	18.9 ± 5.2		103 (69.1)	1189 (55.3)		275 (62.4)	1017 (54.7)	
Yes	1007 (43.8)	18.6 ± 5.3		46 (30.9)	961 (44.7)		166 (37.6)	841 (45.3)	
BMI, kg/m^2^			0.074			0.634			0.094
<25.0 kg/m^2^	2024 (88.0)	18.8 ± 5.3		133 (89.3)	1891 (88.0)		378 (85.7)	1646 (88.6)	
≥3 25.0 kg/m^2^	275 (12.0)	18.2 ± 5.2		16 (10.7)	259 (12.0)		63 (14.3)	212 (11.4)	
Comorbidity			0.435			0.024			0.250
None	2164 (94.1)	18.7 ± 5.3		134 (89.9)	2030 (94.4)		410 (93.0)	1754 (94.4)	
One or more	135 (5.9)	19.1 ± 4.8		15 (10.1)	120 (5.6)		31 (7.0)	104 (5.6)	
S-COVID-19-S ***			0.044			<0.001			<0.001
No	1961 (85.3)	18.7 ± 5.3		100 (67.1)	1861 (86.6)		338 (76.6)	1623 (87.4)	
Yes	338 (14.7)	19.3 ± 5.1		49 (32.9)	289 (13.4)		103 (23.4)	235 (12.6)	
FCoV-19S, Mean ± SD	18.8 ± 5.3			23.0 ± 4.7	18.5 ± 5.2	<0.001	20.4 ± 5.0	18.4 ± 5.2	<0.001
HL, Mean ± SD	36.1 ± 7.1			33.7 ± 6.4	36.3 ± 7.1	<0.001	34.8 ± 6.9	36.4 ± 7.1	<0.001
eHEALS, Mean ± SD	33.1 ± 4.8			32.5 ± 4.8	33.1 ± 4.8	0.172	32.7 ± 5.4	33.2 ± 4.7	0.059
DDL, Mean ± SD	35.8 ± 8.2			33.6 ± 7.5	35.9 ± 8.2	0.001	34.4 ± 8.1	36.1 ± 8.2	<0.001
HES, Mean ± SD	14.6 ± 4.8			13.7 ± 4.6	14.7 ± 4.8	0.013	13.8 ± 4.7	14.8 ± 4.8	<0.001

Abbreviations: FCoV-19S, fear of COVID-19 scale; MH, mental health; HRQoL, health-related quality of life; SD, standard deviation; BMI, body mass index; S-COVID-19-S, suspected coronavirus disease-2019 symptoms; HL, health literacy; eHEALS, eHealth literacy scale; DDL, digital healthy diet literacy; HES, health eating score. *p* values are results of One-Way ANOVA test and Chi-Square test appropriately. * Health care workers were asked whether their mental health got worse, stable, or better during the pandemic as compared to that before the pandemic. ** Health care workers were asked whether their HRQoL got worse, stable, or better during the pandemic as compared to that before the pandemic. *** suspected coronavirus disease-2019 symptoms include fever, cough, dyspnea myalgia, fatigue, sputum production, confusion, headache, sore throat, rhinorrhea, chest pain, hemoptysis, diarrhea, and nausea/vomiting.

**Table 2:** Determinants of fear of COVID-19 among front-line health care workers via bivariate and multivariate linear regression models (*n* = 2299).

Variables	FCoV-19S			
	Bivariate		Multivariate	
	B (95% CI)	*p*	B (95% CI)	*p*
Age, year				
21–40	Reference		Reference	
41–60	0.21 (−0.33, 0.75)	0.444	0.30 (−0.26, 0.85)	0.297
Gender				
Women	Reference		Reference	
Men	−1.10 (−1.56, −0.65)	<0.001	−0.72 (−1.20, −0.24)	0.003
Marital status				
Never married	Reference		Reference	
Ever married	0.87 (0.37, 1.38)	0.001	0.88 (0.36, 1.39)	0.001
Ability to pay for medication				
Very or fairly difficult	Reference		Reference	
Very or fairly easy	−0.63 (−1.06, −0.20)	0.004	−0.28 (−0.72, 0.15)	0.199
Social status				
Low	Reference		Reference	
Middle or high	−0.56 (−1.19, 0.07)	0.084	−0.19 (−0.83, 0.45)	0.554
Type of health care personnel				
Others	Reference		Reference	
Nurse	−0.08 (−0.57, 0.42)	0.765	−0.11 (−0.62, 0.39)	0.659
Doctor	−1.89 (−2.44, −1.33)	<0.001	−1.63 (−2.20, −1.06)	<0.001
Epidemic containment experience				
No	Reference		Reference	
Yes	−0.29 (−0.72, 0.14)	0.188	−0.31 (−0.75, 0.12)	0.158
BMI, kg/m^2^				
<25.0 kg/m^2^	Reference		Reference	
≥25.0 kg/m^2^	−0.60 (−1.27, 0.06)	0.074	−0.31 (−0.98, 0.36)	0.360
Comorbidity				
None	Reference		Reference	
One or more	0.36 (−0.55, 1.28)	0.435	0.22 (−0.69, 1.14)	0.631
S-COVID−19-S *				
No	Reference		Reference	
Yes	0.62 (0.02, 1.23)	0.044	0.51 (−0.09, 1.11)	0.098
HL, One-point increment	−0.08 (−0.11, −0.05)	<0.001		
eHEALS, One-point increment	−0.03 (−0.08, 0.01)	0.125		
DDL, One-point increment	−0.06 (−0.08, −0.03)	<0.001	−0.04 (−0.07, −0.02)	0.001
HES, One-point increment	−0.11 (−0.16, −0.07)	<0.001	−0.10 (−0.15, −0.06)	<0.001

Abbreviations: FCoV-19S, fear of COVID-19 scale; B, unstandardized regression coefficient; CI, confidence interval; BMI, body mass index; S-COVID-19-S, suspected coronavirus disease-2019 symptoms; HL, health literacy; eHEALS, eHealth literacy scale; DDL, digital healthy diet literacy; HES, health eating score. * Suspected coronavirus disease-2019 symptoms include fever, cough, dyspnea, myalgia, fatigue, sputum production, confusion, headache, sore throat, rhinorrhea, chest pain, hemoptysis, diarrhea, and nausea/vomiting.

**Table 3:** Determinants of changes in mental health among front-line health care workers via bivariate and multivariate logistic regression models (*n* = 2299).

Variables	Mental Health Changes *			
	Bivariate		Multivariate	
	OR (95% CI)	*p*	OR (95% CI)	*p*
Age, year				
21–40	Reference		Reference	
41–60	1.39 (0.88, 2.19)	0.162	1.28 (0.79, 2.10)	0.318
Gender				
Women	Reference		Reference	
Men	0.84 (0.60, 1.19)	0.324	0.91 (0.63, 1.33)	0.640
Marital status				
Never married	Reference		Reference	
Ever married	1.39 (0.96, 2.00)	0.078	1.18 (0.80, 1.74)	0.397
Ability to pay for medication				
Very or fairly difficult	Reference		Reference	
Very or fairly easy	1.45 (1.03, 2.03)	0.034	1.36 (0.95, 1.94)	0.094
Social status				
Low	Reference		Reference	
Middle or high	1.55 (1.01, 2.39)	0.045	1.33 (0.84, 2.11)	0.225
Type of health care personnel				
Others	Reference		Reference	
Nurse	1.07 (0.72, 1.60)	0.732	1.04 (0.68, 1.58)	0.872
Doctor	0.78 (0.51, 1.18)	0.239	0.69 (0.44, 1.08)	0.102
Epidemic containment experience				
No	Reference		Reference	
Yes	1.81 (1.27, 2.59)	0.001	1.62 (1.11, 2.35)	0.012
BMI, kg/m^2^				
<25.0 kg/m^2^	Reference		Reference	
≥25.0 kg/m^2^	1.14 (0.67, 1.94)	0.634	1.21 (0.69, 2.12)	0.508
Comorbidity				
None	Reference		Reference	
One or more	0.53 (0.30, 0.93)	0.027	0.65 (0.35, 1.18)	0.154
S-COVID-19-S **				
No	Reference		Reference	
Yes	0.32 (0.22, 0.46)	<0.001	0.36 (0.25, 0.53)	<0.001
HL, One-point increment	1.06 (1.03, 1.09)	<0.001		
eHEALS, One-point increment	1.02 (0.99, 1.06)	0.172		
DDL, One-point increment	1.04 (1.01, 1.06)	0.001	1.02 (1.00, 1.05)	0.029
HES, One-point increment	1.04 (1.01, 1.08)	0.013	1.04 (1.00, 1.07)	0.043

Abbreviations: OR, odds ratio; CI, confidence interval; BMI, body mass index; S-COVID-19-S, suspected coronavirus disease-2019 symptoms; HL, health literacy; eHEALS, eHealth literacy scale; DDL, digital healthy diet literacy; HES, health eating score. * The reference group is ‘worse’, the test group is ‘stable or better’. ** suspected coronavirus disease-2019 symptoms include fever, cough, dyspnea, myalgia, fatigue, sputum production, confusion, headache, sore throat, rhinorrhea, chest pain, hemoptysis, diarrhea, and nausea/vomiting.

**Table 4:** Determinants of changes in health-related quality of life among front-line health care workers via bivariate and multivariate logistic regression models (*n* = 2299).

Variables	HRQoL Changes *			
	Bivariate		Multivariate	
	OR (95% CI)	*p*	OR (95% CI)	*p*
Age, year				
21–40	Reference		Reference	
41–60	1.51 (1.14, 2.01)	0.004	1.53 (1.13, 2.07)	0.006
Gender				
Women	Reference		Reference	
Men	0.72 (0.58, 0.90)	0.003	0.76 (0.60, 0.97)	0.024
Marital status				
Never married	Reference		Reference	
Ever married	1.05 (0.82, 1.34)	0.705	0.88 (0.68, 1.14)	0.347
Ability to pay for medication				
Very or fairly difficult	Reference		Reference	
Very or fairly easy	1.52 (1.23, 1.88)	<0.001	1.43 (1.14, 1.78)	0.002
Social status				
Low	Reference		Reference	
Middle or high	1.59 (1.20, 2.11)	0.001	1.37 (1.02, 1.84)	0.037
Type of health care personnel				
Others	Reference		Reference	
Nurse	1.02 (0.80, 1.30)	0.868	0.95 (0.73, 1.23)	0.708
Doctor	0.94 (0.72, 1.23)	0.638	0.84 (0.63, 1.12)	0.225
Epidemic containment experience				
No	Reference		Reference	
Yes	1.37 (1.11, 1.70)	0.004	1.29 (1.03, 1.61)	0.026
BMI, kg/m^2^				
<25.0 kg/m^2^	Reference		Reference	
≥25.0 kg/m^2^	0.77 (0.57, 1.05)	0.095	0.83 (0.60, 1.15)	0.264
Comorbidity				
None	Reference		Reference	
One or more	0.78 (0.52, 1.19)	0.251	0.90 (0.58, 1.39)	0.636
S-COVID-19-S **				
No	Reference		Reference	
Yes	0.48 (0.37, 0.62)	<0.001	0.53 (0.40, 0.69)	<0.001
HL, One-point increment	1.03 (1.02, 1.05)	<0.001		
eHEALS, One-point increment	1.02 (1.00, 1.04)	0.060		
DDL, One-point increment	1.03 (1.01, 1.04)	<0.001	1.02 (1.01, 1.03)	0.006
HES, One-point increment	1.04 (1.02, 1.07)	<0.001	1.04 (1.02, 1.06)	0.001

Abbreviations: HRQoL, health-related quality of life; OR, odds ratio; CI, confidence interval; BMI, body mass index; S-COVID-19-S, suspected coronavirus disease-2019 symptoms; HL, health literacy; eHEALS, eHealth literacy scale; DDL, digital healthy diet literacy; HES, health eating score. * The reference group is ‘worse’, the test group is ‘stable or better’. ** suspected coronavirus disease-2019 symptoms include fever, cough, dyspnea, myalgia, fatigue, sputum production, confusion, headache, sore throat, rhinorrhea, chest pain, hemoptysis, diarrhea, and nausea/vomiting.

## Data Availability

Data will be available on the reasonable request from the corresponding author.

## Supplementary Materials

The following are available online at https://www.mdpi.com/article/10.3390/nu13082656/s1, Table S1: Spearman correlations (rho) of independent variables (*n* = 2299).

## References

[1] Moitra M., Rahman M., Collins P.Y., Gohar F., Weaver M., Kinuthia J., Rössler W., Petersen S., Unutzer J., Saxena S. (2021). Mental Health Consequences for Healthcare Workers during the COVID-19 Pandemic: A Scoping Review to Draw Lessons for LMICs. Front. Psychiatry.

[2] Li Y., Scherer N., Felix L., Kuper H. (2021). Prevalence of depression, anxiety and post-traumatic stress disorder in health care workers during the COVID-19 pandemic: A systematic review and meta-analysis. PLoS ONE.

[3] Sahebi A., Nejati-Zarnaqi B., Moayedi S., Yousefi K., Torres M., Golitaleb M. (2021). The prevalence of anxiety and depression among healthcare workers during the COVID-19 pandemic: An umbrella review of meta-analyses. Prog. Neuropsychopharmacol. Biol. Psychiatry.

[4] Pappa S., Ntella V., Giannakas T., Giannakoulis V.G., Papoutsi E., Katsaounou P. (2020). Prevalence of depression, anxiety, and insomnia among healthcare workers during the COVID-19 pandemic: A systematic review and meta-analysis. Brain Behav. Immun..

[5] Salari N., Khazaie H., Hosseinian-Far A., Khaledi-Paveh B., Kazeminia M., Mohammadi M., Shohaimi S., Daneshkhah A., Eskandari S. (2020). The prevalence of stress, anxiety and depression within front-line healthcare workers caring for COVID-19 patients: A systematic review and meta-regression. Hum. Resour. Health.

[6] Nguyen P.T.L., Nguyen T.B.L., Pham A.G., Duong K.N.C., Gloria M.A.J., Vo T.V., Vo B.V., Phung T.L. (2021). Psychological Stress Risk Factors, Concerns and Mental Health Support among Health Care Workers in Vietnam during the Coronavirus Disease 2019 (COVID-19) Outbreak. Front. Public Health.

[7] Duffy C.C., Bass G.A., Fitzpatrick G., Doherty E.M. (2020). What can We Learn from the Past? Pandemic Health Care Workers’ Fears, Concerns, and Needs: A Review. J. Patient Saf..

[8] Karlsson U., Fraenkel C.J. (2020). Covid-19: Risks to healthcare workers and their families. BMJ.

[9] Bagcchi S. (2020). Stigma during the COVID-19 pandemic. Lancet Infect. Dis..

[10] Nguyen L.H., Drew D.A., Graham M.S., Joshi A.D., Guo C.G., Ma W., Mehta R.S., Warner E.T., Sikavi D.R., Lo C.H. (2020). Risk of COVID-19 among front-line health-care workers and the general community: A prospective cohort study. Lancet Public Health.

[11] Cag Y., Erdem H., Gormez A., Ankarali H., Hargreaves S., Ferreira-Coimbra J., Rubulotta F., Belliato M., Berger-Estilita J., Pelosi P. (2021). Anxiety among front-line health-care workers supporting patients with COVID-19: A global survey. Gen. Hosp. Psychiatry.

[12] Zaçe D., Hoxhaj I., Orfino A., Viteritti A.M., Janiri L., Di Pietro M.L. (2021). Interventions to address mental health issues in healthcare workers during infectious disease outbreaks: A systematic review. J. Psychiatr. Res..

[13] Pollock A., Campbell P., Cheyne J., Cowie J., Davis B., McCallum J., McGill K., Elders A., Hagen S., McClurg D. (2020). Interventions to support the resilience and mental health of frontline health and social care professionals during and after a disease outbreak, epidemic or pandemic: A mixed methods systematic review. Cochrane Database Syst. Rev..

[14] Greenberg N., Brooks S.K., Wessely S., Tracy D.K. (2020). How might the NHS protect the mental health of health-care workers after the COVID-19 crisis?. Lancet Psychiatry.

[15] Greenberg N., Docherty M., Gnanapragasam S., Wessely S. (2020). Managing mental health challenges faced by healthcare workers during covid-19 pandemic. BMJ.

[16] Apisarnthanarak A., Apisarnthanarak P., Siripraparat C., Saengaram P., Leeprechanon N., Weber D.J. (2020). Impact of anxiety and fear for COVID-19 toward infection control practices among Thai healthcare workers. Infect. Control. Hosp. Epidemiol..

[17] Diver S., Buccheri N., Ohri C. (2021). The value of healthcare worker support strategies to enhance wellbeing and optimise patient care. Future Healthc. J..

[18] Nguyen H.C., Nguyen M.H., Do B.N., Tran C.Q., Nguyen T.T.P., Pham K.M., Pham L.V., Tran K.V., Duong T.T., Tran T.V. (2020). People with suspected COVID-19 symptoms were more likely depressed and had lower health-related quality of life: The potential benefit of health literacy. J. Clin. Med..

[19] Duong T.V., Pham K.M., Do B.N., Kim G.B., Dam H.T.B., Le V.-T.T., Nguyen T.T.P., Nguyen H.T., Nguyen T.T., Le T.T. (2020). Digital Healthy Diet Literacy and Self-Perceived Eating Behavior Change during COVID-19 Pandemic among Undergraduate Nursing and Medical Students: A Rapid Online Survey. Int. J. Environ. Res. Public Health.

[20] Pham K.M., Pham L.V., Phan D.T., Tran T.V., Nguyen H.C., Nguyen M.H., Nguyen H.C., Ha T.H., Dao H.K., Nguyen P.B. (2020). Healthy Dietary Intake Behavior Potentially Modifies the Negative Effect of COVID-19 Lockdown on Depression: A Hospital and Health Center Survey. Front. Nutr..

[21] Nguyen M.H., Pham T.T.M., Nguyen K.T., Nguyen Y.H., Tran T.V., Do B.N., Dao H.K., Nguyen H.C., Do N.T., Ha T.H. (2021). Negative Impact of Fear of COVID-19 on Health-Related Quality of Life Was Modified by Health Literacy, eHealth Literacy, and Digital Healthy Diet Literacy: A Multi-Hospital Survey. Int. J. Environ. Res. Public Health.

[22] Do B.N., Tran T.V., Phan D.T., Nguyen H.C., Nguyen T.T.P., Nguyen H.C., Ha T.H., Dao H.K., Trinh M.V., Do T.V. (2020). Health Literacy, eHealth Literacy, Adherence to Infection Prevention and Control Procedures, Lifestyle Changes, and Suspected COVID-19 Symptoms among Health Care Workers during Lockdown: Online Survey. J. Med. Internet Res..

[23] Quan H., Li B., Couris C.M., Fushimi K., Graham P., Hider P., Januel J.-M., Sundararajan V. (2011). Updating and validating the Charlson comorbidity index and score for risk adjustment in hospital discharge abstracts using data from 6 countries. Am. J. Epidemiol..

[24] Charlson M.E., Pompei P., Ales K.L., MacKenzie C.R. (1987). A new method of classifying prognostic comorbidity in longitudinal studies: Development and validation. J. Chronic Dis..

[25] Editorial Overview of Novel Coronavirus (2019-nCoV). https://bestpractice.bmj.com/topics/en-gb/3000165.

[26] Tran T.V., Nguyen H.C., Pham L.V., Nguyen M.H., Nguyen H.C., Ha T.H., Phan D.T., Dao H.K., Nguyen P.B., Trinh M.V. (2020). Impacts and interactions of COVID-19 response involvement, health-related behaviours, health literacy on anxiety, depression and health-related quality of life among healthcare workers: A cross-sectional study. BMJ Open.

[27] Duong T.V., Aringazina A., Baisunova G., Nurjanah N., Pham T.V., Pham K.M., Truong T.Q., Nguyen K.T., Oo W.M., Su T.T. (2019). Development and validation of a new short-form health literacy instrument (HLS-SF12) for the general public in six Asian countries. Health Lit. Res. Pract..

[28] Duong T.V., Nguyen T.T.P., Pham K.M., Nguyen K.T., Giap M.H., Tran T.D.X., Nguyen C.X., Yang S.-H., Su C.-T. (2019). Validation of the short-form health literacy questionnaire (HLS-SF12) and its determinants among people living in rural areas in Vietnam. Int. J. Environ. Res. Public Health.

[29] HLS-EU Consortium Comparative Report of Health Literacy in Eight EU Member States. The European Health Literacy Project 2009–2012. https://www.healthliteracyeurope.net/hls-eu.

[30] Norman C.D., Skinner H.A. (2006). eHEALS: The eHealth Literacy Scale. J. Med. Internet Res..

[31] Purvis D.L., Lentino C.V., Jackson T.K., Murphy K.J., Deuster P.A. (2013). Nutrition as a component of the performance triad: How healthy eating behaviors contribute to soldier performance and military readiness. US Army Med. Dep. J..

[32] Shams-White M.M., Chui K., Deuster P.A., McKeown N.M., Must A. (2019). Investigating Items to Improve the Validity of the Five-Item Healthy Eating Score Compared with the 2015 Healthy Eating Index in a Military Population. Nutrients.

[33] Ahorsu D.K., Lin C.Y., Imani V., Saffari M., Griffiths M.D., Pakpour A.H. (2020). The Fear of COVID-19 Scale: Development and Initial Validation. Int. J. Ment. Health Addict..

[34] Nguyen H.T., Do B.N., Pham K.M., Kim G.B., Dam H.T.B., Nguyen T.T., Nguyen T.T.P., Nguyen Y.H., Sørensen K., Pleasant A. (2020). Fear of COVID-19 Scale—Associations of Its Scores with Health Literacy and Health-Related Behaviors among Medical Students. Int. J. Environ. Res. Public Health.

[35] Maldonado G., Greenland S. (1993). Simulation study of confounder-selection strategies. Am. J. Epidemiol..

[36] Amatori S., Donati Zeppa S., Preti A., Gervasi M., Gobbi E., Ferrini F., Rocchi M.B.L., Baldari C., Perroni F., Piccoli G. (2020). Dietary Habits and Psychological States during COVID-19 Home Isolation in Italian College Students: The Role of Physical Exercise. Nutrients.

[37] Głąbska D., Guzek D., Groele B., Gutkowska K. (2020). Fruit and Vegetable Intake and Mental Health in Adults: A Systematic Review. Nutrients.

[38] Hoang T.D., Colebunders R., Fodjo J.N.S., Nguyen N.P.T., Tran T.D., Vo T.V. (2021). Well-Being of Healthcare Workers and the General Public during the COVID-19 Pandemic in Vietnam: An Online Survey. Int. J. Environ. Res. Public Health.

[39] Editorial (2021). Health and care workers are owed a better future. Lancet.

[40] López-Moreno M., López M.T.I., Miguel M., Garcés-Rimón M. (2020). Physical and Psychological Effects Related to Food Habits and Lifestyle Changes Derived from Covid-19 Home Confinement in the Spanish Population. Nutrients.

[41] Cawcutt K.A., Starlin R., Rupp M.E. (2020). Fighting fear in healthcare workers during the COVID-19 pandemic. Infect. Control. Hosp. Epidemiol..

[42] Liu S., Yang L., Zhang C., Xu Y., Cai L., Ma S., Wang Y., Cai Z., Du H., Li R. (2021). Gender differences in mental health problems of healthcare workers during the coronavirus disease 2019 outbreak. J. Psychiatr. Res..

[43] Collantoni E., Saieva A.M., Meregalli V., Girotto C., Carretta G., Boemo D.G., Bordignon G., Capizzi A., Contessa C., Nesoti M.V. (2021). Psychological Distress, Fear of COVID-19, and Resilient Coping Abilities among Healthcare Workers in a Tertiary First-Line Hospital during the Coronavirus Pandemic. J. Clin. Med..

[44] Ministry of Health Coronavirus Disease (COVID-19) Outbreak in Vietnam. https://ncov.moh.gov.vn/.

