# Supplementary material for: Impacts of Digital Healthy Diet Literacy and Healthy Eating Behavior on Fear of COVID-19, Changes in Mental Health, and Health-Related Quality of Life among Front-Line Health Care Workers
Source: Nutrients. 2021 Jul 30;13(8):2656. doi: 10.3390/nu13082656 (PMC8398620; doi:10.3390/nu13082656)
Supplement: Supplementary file 1 [file nutrients-13-02656-s001.zip › nutrients-1253381-supplementary.pdf]

Table S1. Spearman correlations (*rho*) of independent variables (n = 2299).

	Age	Gender	Marital status	Ability to pay for medications	Social status	Type of health care personnel	Epidemic containment experience	BMI	Comorbidity	S-COVID-19-S	HL	eHEALS	DDL
Gender	0.028												
Marital status	0.238	-0.030											
Ability to pay for medications	0.019	-0.004	-0.001										
Social status	0.064	-0.052	0.056	0.178									
Type of health care personnel	-0.056	-0.091	0.026	-0.093	-0.165								
Epidemic containment experience	0.131	0.023	0.102	-0.005	0.019	-0.034							
BMI	0.041	0.229	0.066	0.050	0.015	-0.044	0.050						
Comorbidity	0.107	0.028	0.056	-0.022	-0.043	-0.028	0.011	0.050					
S-COVID-19-S	-0.010	0.038	-0.024	-0.035	-0.054	-0.022	-0.027	0.048	0.152				
HL	0.025	0.075	0.001	0.128	0.048	-0.095	0.134	0.037	-0.003	-0.076			
eHEALS	-0.017	0.123	0.024	0.067	0.004	-0.051	0.080	0.057	-0.009	-0.059	**0.450**		
DDL	0.014	0.053	0.020	0.077	0.053	-0.004	0.072	0.028	-0.027	-0.102	**0.680**	**0.378**	
HES	0.012	-0.040	0.046	0.050	0.020	-0.034	0.041	-0.030	0.039	-0.037	0.109	0.048	0.084

Abbreviations: BMI, body mass index; S-COVID-19-S, suspected coronavirus disease-2019 symptoms; HL, health literacy; eHEALS, eHealth literacy scale; DDL, digital healthy diet literacy; HES, health eating score.
